# Supplementary figures and images for: Evidence linking atopy and staphylococcal superantigens to the pathogenesis of lymphomatoid papulosis, a recurrent CD30+ cutaneous lymphoproliferative disorder
Source: PLoS One. 2020 Feb 12;15(2):e0228751. doi: 10.1371/journal.pone.0228751 (PMC7015403; doi:10.1371/journal.pone.0228751)

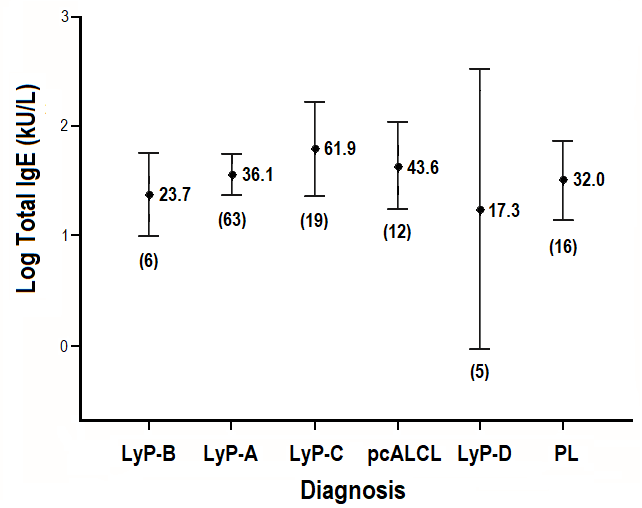

Supplement: S1 Fig — Number patients in parentheses. (DOCX) [file pone.0228751.s001.docx]

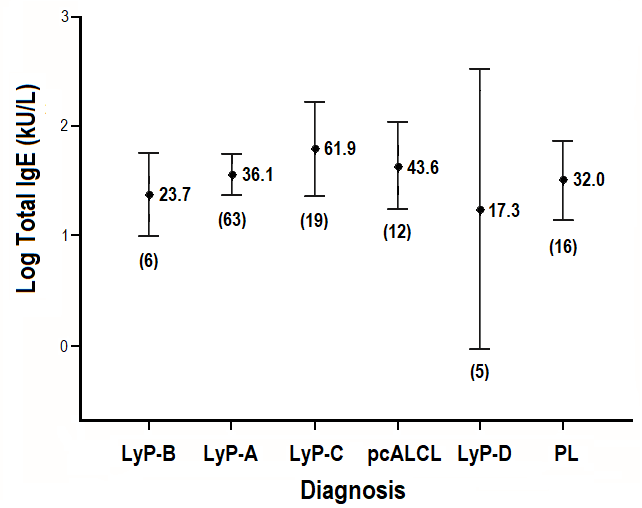

Supplement: S2 Fig — (DOCX) [file pone.0228751.s002.docx]
