# Supplementary material for: Evidence linking atopy and staphylococcal superantigens to the pathogenesis of lymphomatoid papulosis, a recurrent CD30+ cutaneous lymphoproliferative disorder
Source: PLoS One. 2020 Feb 12;15(2):e0228751. doi: 10.1371/journal.pone.0228751 (PMC7015403; doi:10.1371/journal.pone.0228751)
Supplement: S1 Table — (DOCX) [file pone.0228751.s003.docx]

| Diagnosis | No. Aero-IgE < 0.35‡ | No. Aero-IgE ≥ 0.35‡ | P-value* |
| --- | --- | --- | --- |
| All CD30CLPD† | 16 | 15 (48%) |  |
| No PHx atopy | 11 | 13 | 0.394 |
| Hx PHx atopy | 5 | 2 |  |
| All LyP-A and C | 14 | 14 (50%) |  |
| No PHx atopy | 10 | 12 | 0.648 |
| Hx PHx atopy | 4 | 2 |  |

Abbreviations: CD30CLPD, primary cutaneous CD30+ lymphoproliferative disorder, LyP, lymphomatoid papulosis; No., number patients in cohort.

‡ Aero-IgE concentration in kUa/L.

† Group includes LyP-A (n= 19), LyP-C (n= 9) and primary cutaneous CD30+ anaplastic large cell lymphoma (n= 3).

* Fisher’s exact test.
