# Supplementary material for: Evidence linking atopy and staphylococcal superantigens to the pathogenesis of lymphomatoid papulosis, a recurrent CD30+ cutaneous lymphoproliferative disorder
Source: PLoS One. 2020 Feb 12;15(2):e0228751. doi: 10.1371/journal.pone.0228751 (PMC7015403; doi:10.1371/journal.pone.0228751)
Supplement: S2 Table — (DOCX) [file pone.0228751.s004.docx]

| Supplemental Group | No. | IgE-t Median (range) | IgE-t GM (95% CI) | KW* | t-test* |
| --- | --- | --- | --- | --- | --- |
| All CD30CLPD | 105 | 37.0 (1.4-11146) | 38.3 (27.3-53.8) |  |  |
| No PHx atopy | 64 | 28.5 (1.4-4660) | 27.3 (17.8-41.7) | 0.022 | 0.013 |
| PHx atopy | 41 | 57.0 (4.0-11146) | 65.3 (37.9-113) |  |  |
| All LyP | 94 | 38.0 (1.4-11146) | 37.1(25.7-53.6) |  |  |
| No PHx atopy | 55 | 30.0 (1.4-4660) | 26.1 (16.2-42.0) | 0.040 | 0.023 |
| PHx atopy | 39 | 52.0 (4.0-11146) | 61.0 (34.7-107) |  |  |
| All LyP-B | 6 | 34.0 (6.0-46) | 23.7 (9.9-56.5) |  |  |
| No PHx atopy | 4 | 43.5 (26.0-46) | 38.8 (25.3-59.5) | 0.064 | 0.131 |
| PHx atopy | 2 | 9.5 (6.0-13) | 8.8 |  |  |
| All LyP-A | 62 | 38.0 (1.4-4660) | 35.9 (23.4-55.8) |  |  |
| No PHx atopy | 37 | 24.0 (1.4-4660) | 22.0 (12.4-39.0) | 0.007 | 0.005 |
| PHx atopy† | 25 | 61.0 (4.3-1633) | 73.9 (39.2-140) |  |  |
| All LyP-C | 20 | 40.0 (3.0-11146) | 56.2 (21.6-147) |  |  |
| No PHx atopy | 10 | 41.5 (3.0-980) | 58.8 (15.8-219) | 0.597 | 0.925 |
| PHx atopy‡ | 10 | 29.5 (4.0-11146) | 53.7 (10.1-287) |  |  |
| All LyP-D | 6 | 31.5 (2.0-478) | 21.1 (2.1-208) |  |  |
| No PHx atopy | 4 | 4.5 (2.0-478) | 11.5 (0.2-640) | 0.355 | 0.244 |
| PHx atopy | 2 | 73.5 (57.0-90) | 71.6 |  |  |
| All pcALCL | 11 | 30.0 (7.0-469) | 50.3 (19.5-130) |  |  |
| No PHx atopy | 9 | 24.0 (7.0-469) | 35.3 (12.9-96.7) | 0.099 | 0.002 |
| PHx atopy | 2 | 245.5 (232-259) | 245 |  |  |
| All PL | 16 | 32.5 (1.4-343) | 32.0 (13.9-73.7) |  |  |
| No PHx atopy | 11 | 34.0 (1.4-343) | 35.2 (11.3-110) | 0.692 | 0.714 |
| PHx atopy | 5 | 22.0 (6.0-192) | 25.9 (4.6-147) |  |  |

Abbreviations: CD30CLPD, primary cutaneous CD30+ lymphoproliferative disorder; LyP, lymphomatoid papulosis; pcALCL, primary cutaneous anaplastic large cell lymphoma; PL, pityriasis lichenoides; No., number patients in cohort; IgE-t, total serum IgE in kU/L; GM, geometric mean and 95% confidence interval.

* P-values for Kruskal-Wallis and t-test of 2 independent samples.

† Includes one patient with active atopic dermatitis.

‡ Includes one patient with active atopic dermatitis and one with nonspecific eczema.
