# Supplementary material for: Evidence linking atopy and staphylococcal superantigens to the pathogenesis of lymphomatoid papulosis, a recurrent CD30+ cutaneous lymphoproliferative disorder
Source: PLoS One. 2020 Feb 12;15(2):e0228751. doi: 10.1371/journal.pone.0228751 (PMC7015403; doi:10.1371/journal.pone.0228751)
Supplement: S3 Table — (DOCX) [file pone.0228751.s005.docx]

| Diagnosis | Aero-IgE† | No. | IgE-t Median (range) | IgE-t GM  (95% CI) | KW* | t-test* | Dunnett** |
| --- | --- | --- | --- | --- | --- | --- | --- |
| All CD30CLPD | < 0.35 | 16 | 53.5 (4.3-927) | 55.4 (22.4-137) | 0.502 | 0.456 | 0.011 |
|  | ≥ 0.35 | 15 | 53.7 (20.7-598) | 81.6 (44.5-150) |  |  |  |
| LyP-A | < 0.35 | 9 | 70.8 (6.6-927) | 75.6 (20.6-277) | 0.870 | 0.899 | 0.002 |
|  | ≥ 0.35 | 10 | 50.5 (20.7-598) | 82.6 (33.2-206) |  |  |  |
| LyP-C | < 0.35 | 5 | 62.8 (11.7-912) | 73.9 | 1.0 | 0.994 | 0.027 |
|  | ≥ 0.35 | 4 | 50.6 (46.7-256) | 74.3 (19.9-277) |  |  |  |
| pcALCL | < 0.35 | 2 | 7.4 (4.3-10.4) | 7.0 | ---- | ---- | ---- |
|  | ≥ 0.35 | 1 | 104 | 104 | ---- | ---- | ---- |
| Control | < 0.35 | 52 | 14.8 (2.0-187) | 14.1 (10.0-19.9) | ---- | ---- | ---- |

Abbreviations: CD30CLPD, primary cutaneous CD30+ lymphoproliferative disease; LyP, lymphomatoid papulosis; pcALCL, primary cutaneous anaplastic large cell lymphoma; PL, pityriasis lichenoides; No., number patients in cohort; IgE-t, total serum IgE in kU/L; GM, geometric mean and 95% confidence interval.

† Aero-IgE concentration in kUa/L.

* Comparison of IgE-t in disease subsets with aero-IgE < 0.35 versus ≥ 0.35 kUa/L by Kruskal-Wallis or t-test of 2 independent samples.

** Comparison of IgE-t in disease subsets with aero-IgE < 0.35 with control by Dunnett’s t-test.
