# Supplementary material for: Evidence linking atopy and staphylococcal superantigens to the pathogenesis of lymphomatoid papulosis, a recurrent CD30+ cutaneous lymphoproliferative disorder
Source: PLoS One. 2020 Feb 12;15(2):e0228751. doi: 10.1371/journal.pone.0228751 (PMC7015403; doi:10.1371/journal.pone.0228751)
Supplement: S4 Table — (DOCX) [file pone.0228751.s006.docx]

| Supplemental Group | No. | IgE-t Median (range) | IgE-t GM (95% CI) |
| --- | --- | --- | --- |
| All LyP | 93 | 39.0 (1.4-11146) | 37.7 (26.0-54.6) |
| LyP-B | 6 | 34.0 (6.0-46) | 23.7 (9.9-56.5) |
| LyP-A | 63 | 39.0 (1.4-4660) | 36.1 (23.4-55.8) |
| LyP-C | 19 | 40.0 (3.0-11146) | 61.9 (23.0-167) |
| LyP-D | 5 | 6.0 (2.0-478) | 17.3 (0.9-331) |
| pcALCL | 12 | 30.0 (7.0-469) | 43.6 (17.5-108) |
| PL | 16 | 32.5 (1.4-343) | 32.0 (13.9-73.7) |

Abbreviations: LyP, lymphomatoid papulosis; pcALCL, primary cutaneous anaplastic large cell lymphoma; PL, pityriasis lichenoides; No., number patients in cohort; IgE-t, total serum IgE (kU/L); GM, geometric mean and 95% confidence interval.
