# Supplementary material for: Evidence linking atopy and staphylococcal superantigens to the pathogenesis of lymphomatoid papulosis, a recurrent CD30+ cutaneous lymphoproliferative disorder
Source: PLoS One. 2020 Feb 12;15(2):e0228751. doi: 10.1371/journal.pone.0228751 (PMC7015403; doi:10.1371/journal.pone.0228751)
Supplement: S5 Table — (DOCX) [file pone.0228751.s007.docx]

| No. CD30+ Cells | No. | IgE-t Median (range) | IgE-t GM (95% CI) | KW* | ANOVA* |
| --- | --- | --- | --- | --- | --- |
| < 5% | 30 | 23.5 (1.4-1633) | 26.7 (13.2-54.3) | 0.516 | 0.607 |
| 5-19% | 26 | 47.5 (1.4-4660) | 49.8 (23.9-103) |  |  |
| 20-49% | 20 | 38.0 (1.4-950) | 40.8 (17.6-94.2) |  |  |
| ≥ 50% | 17 | 28.0 (3.0-11146) | 47.0 (16.8-132) |  |  |
| All CD30CLPD | 93 | 35.0 (1.4-11146) | 38.6 (26.3-56.6) |  |  |

Abbreviations: CD30CLPD, primary cutaneous CD30+ lymphoproliferative disorder; No., number patients in cohort; IgE-t, total serum IgE (kU/L); GM, geometric mean and 95% confidence interval.

* Kruskal-Wallis and one way analysis of variance of independent samples.
