# Supplementary material for: Evidence linking atopy and staphylococcal superantigens to the pathogenesis of lymphomatoid papulosis, a recurrent CD30+ cutaneous lymphoproliferative disorder
Source: PLoS One. 2020 Feb 12;15(2):e0228751. doi: 10.1371/journal.pone.0228751 (PMC7015403; doi:10.1371/journal.pone.0228751)
Supplement: S6 Table — (DOCX) [file pone.0228751.s008.docx]

|  | No. | SSAg-IgE  ≥ 0.1 | P* | SSAg-IgE  ≥ 0.35 | P* | TSST1-IgE  ≥ 0.35 | P* |
| --- | --- | --- | --- | --- | --- | --- | --- |
| Diagnosis |  |  |  |  |  |  |  |
| LyP-A | 19 | 9 (47%) | 0.434 | 5 (26%) | 0.927 | 4 (21%) | 0.858 |
| LyP-C | 9 | 2 (22%) |  | 2 (22%) |  | 2 (22%) |  |
| pcALCL | 3 | 1 (33%) |  | 1 (33%) |  | 0 (0%) |  |
| CD30+ cells |  |  |  |  |  |  |  |
| < 5% | 9 | 4 (44%) | 0.121 | 4 (44%) | 0.121 | 4 (44%) | 0.131 |
| 5-19% | 7 | 2 (29%) |  | 0 (0%) |  | 0 (0%) |  |
| 20-49% | 8 | 4 (50%) |  | 3 (38%) |  | 2 (25%) |  |
| ≥ 50% | 5 | 0 (0%) |  | 0 (0%) |  | 0 (0%) |  |
| ND | 2 | 2 (100%) |  | 0 (0%) |  | 0 (0%) |  |
| Control | 52 | 6 (12%) | ---- | 3 (6%) | ---- | 3 (6%) | ---- |

Abbreviations: LyP, lymphomatoid papulosis; pcALCL, primary cutaneous anaplastic large cell lymphoma; No., number patients in cohort; GM, geometric mean and 95% confidence interval.

* P-values determined by Chi-square exact test.
