# Supplementary material for: Evidence linking atopy and staphylococcal superantigens to the pathogenesis of lymphomatoid papulosis, a recurrent CD30+ cutaneous lymphoproliferative disorder
Source: PLoS One. 2020 Feb 12;15(2):e0228751. doi: 10.1371/journal.pone.0228751 (PMC7015403; doi:10.1371/journal.pone.0228751)
Supplement: S7 Table — (DOCX) [file pone.0228751.s009.docx]

| Diagnosis | No. | IgE-t Median (range) | IgE-t GM (95% CI) | KW* | ANOVA* |
| --- | --- | --- | --- | --- | --- |
| All CD30CLPD |  |  |  |  |  |
| SCS never | 110 | 35.7 (1.4-11146) | 37.1 (27.1-50.7) | 0.034 | 0.050 |
| SCS prior | 19 | 74.4 (1.4-11146) | 55.5 (23.0-134) |  |  |
| SCS current | 5 | 302.0 (16.0-927) | 224.3 (32.4-1552) |  |  |
| All LyP |  |  |  |  |  |
| SCS never | 99 | 36.2 (1.41-11146) | 36.2(25.9-50.5) | 0.018 | 0.037 |
| SCS prior | 17 | 78.0 (1.41-683) | 71.4 (28.1-181) |  |  |
| SCS current | 5 | 302.0 (16.0-927) | 224 (32.4-1552) |  |  |
| LyP-A |  |  |  |  |  |
| SCS never | 63 | 35.3 (1.4-4660) | 37.0 (24.6-55.8) | 0.088 | 0.074 |
| SCS prior | 11 | 62.6 (1.4-683) | 39.4 (10.5-148) |  |  |
| SCS current | 5 | 302 (16.0-927) | 224 (32.4-1552) |  |  |

Abbreviations: CD30CLPD, primary cutaneous CD30+ lymphoproliferative disorder; LyP, lymphomatoid papulosis; No., number patients in cohort; total serum IgE (kU/L); GM, geometric mean and 95% confidence interval.

* Differences in IgE-t levels tested by Kruskal-Wallis and one-way analysis of variance tests
