# Supplementary material for: Evidence linking atopy and staphylococcal superantigens to the pathogenesis of lymphomatoid papulosis, a recurrent CD30+ cutaneous lymphoproliferative disorder
Source: PLoS One. 2020 Feb 12;15(2):e0228751. doi: 10.1371/journal.pone.0228751 (PMC7015403; doi:10.1371/journal.pone.0228751)
Supplement: S8 Table — (DOCX) [file pone.0228751.s010.docx]

| Diagnosis | No. | IgE-t Median (range) | IgE-t GM (95% CI) | KW* | ANOVA* |
| --- | --- | --- | --- | --- | --- |
| All CD30CLPD |  |  |  |  |  |
| Non-Smoker | 39 | 38.3 (1.4-683) | 33.2 (20.3-54.2) | 0.952 | 0.560 |
| Prior Smoker | 28 | 29.2 (4.0-11146) | 53.0 (23.0-122) |  |  |
| Current Smoker | 40 | 39.5 (1.4-927) | 38.9 (22.7-66.5) |  |  |
| All LyP |  |  |  |  |  |
| Non-Smoker | 37 | 43.0 (1.41-683) | 36.2 (21.9-59.7) | 0.999 | 0.583 |
| Prior Smoker | 25 | 25.0 (4.0-11146) | 52.4 (21.3-129) |  |  |
| Current Smoker | 36 | 39.5 (1.41-927) | 37.0 (20.9-65.5) |  |  |

Abbreviations: CD30CLPD, primary cutaneous CD30+ lymphoproliferative disorder; LyP, lymphomatoid papulosis; No., number patients in cohort; total serum IgE (kU/L); GM, geometric mean and 95% confidence interval.

* Differences in IgE-t levels tested by Kruskal-Wallis and one-way analysis of variance tests.
